# The Effect of Sterilization on the Characteristics of Silk Fibroin Nanoparticles

**DOI:** 10.3390/polym14030498

**Published:** 2022-01-26

**Authors:** María Alejandra Asensio Ruiz, Marta G. Fuster, Teresa Martínez Martínez, Mercedes G. Montalbán, José Luis Cenis, Gloria Víllora, Antonio Abel Lozano-Pérez

**Affiliations:** 1 Unidad de Radiofarmacia, Hospital Clínico Universitario Virgen de la Arrixaca, 30120 Murcia, Spain; mariaa.asensio@carm.es (M.A.A.R.); mteresa.martinez5@carm.es (T.M.M.); 2 Instituto Murciano de Investigación Biosanitaria (IMIB)-Arrixaca, 30120 Murcia, Spain; 3 Chemical Engineering Department, Faculty of Chemistry, Regional Campus of International Excellence “Campus Mare Nostrum”, University of Murcia, 30071 Murcia, Spain; marta.g.f@um.es (M.G.F.); gvillora@um.es (G.V.); 4 Departamento de Biotecnología, Genómica y Mejora Vegetal, Instituto Murciano de Investigación y Desarrollo Agrario y Medioambiental (IMIDA), 30150 Murcia, Spain; josel.cenis@carm.es

**Keywords:** biopolymer, nanoparticles, sterilization, autoclave, gamma irradiation, biomedical applications

## Abstract

In recent years, silk fibroin nanoparticles (SFNs) have been consolidated as drug delivery systems (DDSs) with multiple applications in personalized medicine. The design of a simple, inexpensive, and scalable preparation method is an objective pursued by many research groups. When the objective is to produce nanoparticles suitable for biomedical uses, their sterility is essential. To achieve sufficient control of all the crucial stages in the process and knowledge of their implications for the final characteristics of the nanoparticles, the present work focused on the final stage of sterilization. In this work, the sterilization of SFNs was studied by comparing the effect of different available treatments on the characteristics of the nanoparticles. Two different sterilization methods, gamma irradiation and autoclaving, were tested, and optimal conditions were identified to achieve the sterilization of SFNs by gamma irradiation. The minimum irradiation dose to achieve sterilization of the nanoparticle suspension without changes in the nanoparticle size, polydispersity, or Z-potential was determined to be 5 kiloGrays (kGy). These simple and safe methods were successfully implemented for the sterilization of SFNs in aqueous suspension and facilitate the application of these nanoparticles in medicine.

## 1. Introduction

In recent years, silk fibroin nanoparticles (SFNs) have been extensively studied and are considered as promising drug delivery systems (DDSs) with multiple applications in the era of personalized medicine [1]. When regenerated in the form of nanoparticles, silk fibroin (SF), an “FDA-Approved biopolymer”, can act as an excellent vehicle, efficiently transporting drugs with low bioavailability to target tissues [1,2]. In contrast to synthetic polymers, SFNs obtained from domesticated silkworms present interesting characteristics, such as the high availability of raw materials for their preparation, a wide range of preparation methods, biocompatibility, biodegradability, and functionalization capacity. These characteristics make them ideal for use as DDSs [3]. SF-based DDSs are able to stabilize not only sensitive small drugs, but also large biological molecules such as nucleic acids, peptides, or proteins, enhancing their shelf lives and controlling their release, either by physical adsorption or encapsulation [4].

The design of a simple, inexpensive, and scalable preparation method is an objective pursued by many researchers. The number of available preparation methods for SFNs have increased considerably since the study published by Zhang et al. in 2007 [5]. A broad spectrum of manufacturing strategies have been used to generate *Bombyx mori* silk nanoparticles (reviewed in [6,7,8,9]), and can be classified into two groups according to the approach: bottom-up or top-down. Among the bottom-up approaches, the most commonly used are the desolvation of an aqueous or ionic liquid silk fibroin solution in organic polar solvents [5,10], salting out [11], or the laminar jet break-up process [12]. Comparatively, top-down methods, such as ball-milling, can be an interesting and alternative approach, although the polydispersity of the particles is higher than those observed in the bottom-up approaches [7].

Thus, knowledge and control of all the crucial stages of the process and their implications when it comes to the final characteristics of the nanoparticles have not only led the efforts of our research group, but also that of other researchers who have worked with SFNs in recent years. On one hand, although the effect on the characteristics of the nanoparticles of several parameters involved in the production of the SFNs have been previously studied, including the degumming method [13], the composition of the precipitating agent [2,5,14], the influence of the pH [15,16], or the ionic strength [11], there is still a lack of information about how sterilization affects SFN performance.

On the other hand, it is well known that protein-based biomaterials present different responses to different sterilization methods. Thus, due the complexity of protein structures, heat or irradiation may result in the loss of their physical or biological properties [17]. In recent years, the effects of sterilization methods of SF biomaterials have been studied on solution [18,19,20,21] and regenerated solid biomaterials (films, electrospun mats, scaffolds) [22,23,24]. However, the specific effects of different methods of sterilization on the final characteristics of the nanoparticles have not yet been addressed.

The most commonly used sterilization methods are based on exposition to moist heat with high pressure (autoclaving), dry heat, gamma irradiation, and exposure to either ethylene oxide or hydrogen peroxide plasma. SF biomaterials have been previously sterilized via autoclaving, exposure to ethylene oxide, UV and gamma irradiation, and immersion in ethanol or methanol solutions. A complete summary of findings relating to the effects of different sterilization techniques on the properties of silk fibroin protein solution and lyophilized SF scaffolds were reviewed by Rnjack-Kovacina et al. [22]. Autoclaving results in the significant fragmentation of the fibroin chains, with a reduction in their molecular weight, which produces different effects on silk biomaterials depending on their physical state. While autoclaving favours SF aggregation in aqueous solutions, when the treatment is applied to solid SF regenerated as 3D scaffolds, it produces an increase in the compressive modulus and the degradation rate. Similarly, gamma irradiation produces potential protein damage and cross-link formation in the scaffolds [22].

According to the requirements of pharmacopeias, the sterility of parenteral administered compounds is an indispensable prerequisite, and the selection of the optimal sterilization method is guided by the Guideline on the sterilisation of the medicinal product, active substance, excipient and primary container, provided by the European Medicines Agency [25]. In case of aqueous based formulations, sterilization by moist heat at 121 °C for 15 min is the method of choice.

Sterilization by gamma irradiation is a simple, safe, and effective technique commonly used in the pharmaceutical industry that consists of the controlled exposure of a product to ionizing radiation emitted through an isotopic source. The effect of this irradiation when sterilizing nanoparticles not only depends on the radiation dose applied but also on the nanoparticle reactivity. The exposure of co-polymeric nanoparticles composed of a mixture of poly-(ε-caprolactone) (PCL) and poly-(D, L-lactic-co-glycolic) acid (PLGA) to gamma irradiation at low doses (5 and 10 kGy) in the presence of the cryo-protectant polyvinyl alcohol (PVA) slightly modified the mean particle size and zeta potential of the particles. Exposure to gamma irradiation did not significantly affect the chemical properties of these polymers [26]. However, it is noted that gamma irradiation of chitosan hydrogel nanoparticles at doses of 8, 13, and 25 kGy resulted in changes in their structure with the formation of visible sediments [27]. Indeed, when silver nanoparticles were irradiated with doses commonly used in the pharmaceutical industry (15, 25, and 50 kGy), dramatic changes in particle size and morphology of the polymer were observed [28].

Thus, the aim of the present study is the ascertainment of the effects of methods of sterilization on the characteristics and the performance of SFNs, focused, for accessibility reasons, on the comparison between autoclaving and gamma irradiation. Additionally, it is hoped that the results of this study increase awareness of this promising biopolymeric carrier which can act as an efficient DDS.

## 2. Materials and Methods

**Chemicals**. All the chemicals and solvents used were purchased from Merck (Madrid, Spain). Ultrapure water (18.2 MΩ·cm^−1^) from a Purelab Flex 2 (ELGA, High Wycombe, UK) was used throughout.

### 2.1. Preparation of the Silk Fibroin Solution

White silk cocoons were obtained from *Bombyx mori* silkworms fed with fresh natural *Morus alba L*. leaves in the IMIDA’s sericulture facilities (Murcia, Spain). White cocoons were opened using scissors and the chrysalides were removed prior to being degummed in a boiling aqueous solution of Na_2_CO_3_ 0.05 M for 120 min in order to efficiently remove the sericin and produce smaller nanoparticles with the highest surface charge density [13]. The SF fibres were further rinsed with ultrapure water and dried at room temperature overnight. Then, SF was dissolved at 10% (*w*/*v*) in LiBr 9.3 M for 3 h at 65 °C, as previously described [29]. The Ambar-like SF solution was then filtered in order to remove residual fibres or dust particles and stored at 4 °C until use.

### 2.2. Preparation of the Silk Fibroin Nanoparticles (SFN)

SFNs were prepared via nanoprecipitation in methanol, adapting our previously described method [30]. Briefly, SF was dissolved at 10% (*w*/*v*) in the solvent mixture CaCl_2_/ethanol/H_2_O (1:2:8, molar ratio), also known as Ajisawa’s reagent [31]. The hydro-alcoholic SF solution was then filtered and dialyzed against ultrapure water using a cellulose semipermeable membrane(SnakeSkin^TM^ Dialysis Tubing (Part No. 88244), Pierce Biotechnology, Rockford, IL, USA) to obtain the SF aqueous solution at 2% (*w*/*v*), which is used for nanoparticle preparation by slowly dripping it into vigorously stirred methanol. After a few drops, a milky suspension appeared and after the complete addition of the silk, the nanoparticle suspension was stirred for further 2 h to complete the transition to β-sheet. Then, the resulted nanoparticle suspension was recovered using centrifugation at 8000× *g* for 30 min at 8 °C (Eppendorf Centrifuge 5810R equipped with an F-34-6-38 rotor, Eppendorf AG, Hamburg, Germany)). The particle precipitate was repeatedly washed (3×) with water in order to remove the methanol, and then dispersed in ultrapure water by using high power ultrasounds for 1 min at 10% of amplitude in a Branson Digital Sonifier SFX 550 equipped with a 1/8” tapered microtip (Branson Ultrasonics Corp, Danbury, CT, USA). Finally, the concentration of nanoparticles was measured by weighting dried replicates of known volumes of the SFN suspension (*n* = 3), adjusted to 10 mg/mL with ultrapure water and stored at 4 °C until use.

### 2.3. Nanoparticle Sterilization

In order to assess the effect of different sterilization techniques on SFNs, two of the aforementioned methods were tested [22]. The SFN suspensions at 10 mg/mL (5 mL/test in 15 mL plastic vials) were then sterilized prior to their characterization either by autoclaving them (SFN-A) at 121 °C for 20 min under a high pressure saturated steam cycle for liquids (Autoclave Presoclave II, J.P. Selecta, Barcelona, Spain) or via gamma irradiation (SFN-I) by exposing them to a source of ^137^Cesium (at 5.8 Gy/min) at doses of 1, 2.5, 5, and 10 kGy (Biobeam GM 8000, Gamma-Service Medical GmbH, Leipzig, Germany).

### 2.4. Nanoparticle Characterization

The characterization of the nanoparticles was performed using common techniques, such as field emission scanning electron microscopy (FESEM), dynamic light scattering (DLS), and attenuated total reflectance-Fourier transformed infrared spectroscopy (ATR-FTIR).

The morphology of the nanoparticles was observed by using a FESEM APREO S (Thermo Fisher Scientific Inc., Waltham, MA, USA). An aliquot (10 µL) of an aqueous suspension of nanoparticles (10 µg/mL) was dropped onto a clean glass wafer before drying overnight and finally sputtered with platinum for 5 min resulting in a 5.13 nm coating thickness (Leica, EM ACE600, Leica Microsystems Inc., Concord, ON, Canada). The morphology was studied by collecting images at a magnification of 50,000× using a T3 detector in the immersion mode (current 0.10 nA, accelerating voltage of 5.00 kV, WD = 4.5–5.0 mm).

The size distribution and superficial charge density of nanoparticles were determined by DLS using a Zetasizer Nano ZSP instrument (Malvern Panalytical Ltd., Malvern, UK) following the procedure described previously [30].

ATR-FTIR analysis of the nanoparticles was performed in order to detect possible structural changes in the SF after sterilization. Infrared spectra of ~2 mg of the freeze-dried samples were acquired on a Nicolet iS5 spectrometer equipped with an iD5 ATR accessory (Nicolet, Thermo Scientific, Waltham, MA, USA) controlled by OMNIC software ver. 9.7.39. Measurement conditions were set as previously described [30].

### 2.5. Stability Assays

The effects of the solvent and the temperature on the stability of the sterilized SFN suspensions were studied in two independent assays for short-term and long-term storage. One set of conditions included the suspension of nanoparticles at 1 mg/mL, prepared either in ultrapure water or buffered phosphate saline solution (PBS 1×), and refrigerated at 4 ± 2 °C or incubated at 37 ± 1 °C until their characterization by DLS after 0, 7, 15, and 30 days of incubation. Alternatively, the nanoparticle suspensions were stored for 180 days at 4 °C and characterized by DLS.

### 2.6. Microbiological Assays

The sterility of the nanoparticles was assessed by the Department of Microbiology at the University Hospital Virgen de la Arrixaca (Murcia, Spain). Samples of 100 µL of the suspension of nanoparticles were directly inoculated, under aseptic conditions, into BACT/ALERT PF PLUS culture bottles (Ref. 410853, BioMérieux España S.A, Madrid, Spain) and incubated at 32.5 ± 2.5 °C for 14 days while aerobic and facultative anaerobic microorganisms were looking for by using the BACT/ALERT^®^ 3D Microbial Detection System (BioMérieux España S.A., Madrid, Spain). Negative and positive controls were included. Positive bottles were subcultured into 5% sheep’s blood agar. Samples of the grown colonies were then identified in a MALDI-TOF (Matrix Assisted Laser Desorption Ionization Time-of-Flight) mass spectrometry microbial identification system (VITEK^®^ MS, BioMérieux España S.A, Madrid, Spain) including the VITEK^®^ MS IVD and VITEK^®^ MS RUO, for the clinically relevant species database and the broad research database for microorganisms, respectively.

### 2.7. Citoxicity/Citocompatibility Assays

The cytotoxicity of sterilized and non-sterilized SFNs on L929 cells was assessed by alamarBlue Cell Viability Assay Reagent (Boster Biological Technology, Pleasanton, CA, USA). Cells were seeded in 96-well plates at a concentration of 5 × 10^3^ cells/well. Twenty-four hours later, the cells were fed with fresh culture medium supplemented with different final concentrations of nanoparticles (0.05, 0.10, 0.25, and 0.50 mg/mL) for 48 h. Growth medium without nanoparticles was used as a control. Then, the medium was removed and the AlamarBlue^®^ assay (Thermo Fisher Scientific, Waltham, MA, USA) was performed following the manufacturer’s protocol. Fluorescence was measured in a FLUOstar Omega Microplate Reader (BMG LABTECH GmbH, Freiburg, Germany) spectrophotometer using an excitation wavelength of 530 nm and an emission wavelength of 590 nm. Each sample was tested in three independent sets.

### 2.8. Statistical Analysis

All experiments were performed at least in triplicate and results are presented as mean value ± standard deviation (SD). Statistical analysis of the experimental results was performed by using one-way analysis of variance (ANOVA) with SPSS 16 software. Comparisons between groups were made by performing a Student’s test. The reported *p*-values were considered statistically significant at *p* < 0.05.

## 3. Results

**Figure 1:**
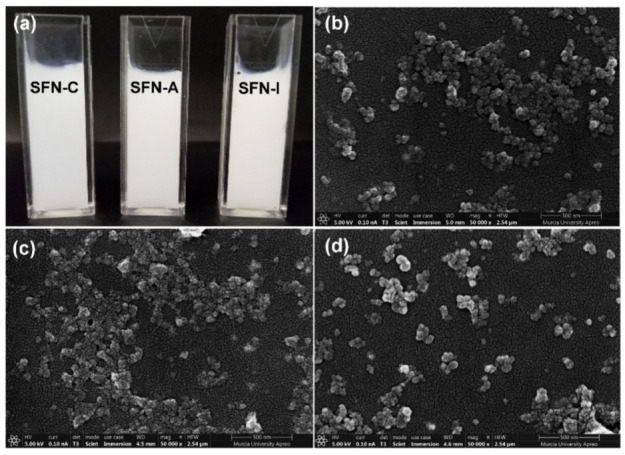
(**a**) Photograph of the non-sterilized (SFN-C), autoclaved (SFN-A), and gamma irradiated at 5 kGy (SFN-I) nanoparticle suspensions at 10 mg/mL in ultrapure water; FESEM images of: (**b**) SFN-C; (**c**) SFN-A, and (**d**) SFN-I. Scale bar = 500 nm.

In a preliminary visual inspection, the sterilized nanoparticle suspensions, either via autoclaving (SFN-A) or gamma irradiation (SFN-I), were apparently indistinguishable from non-sterilized SFNs (SFN-C). Neither changes in the colour or aspect of the suspensions, nor aggregation or sedimentation were observed in the sterilized samples, as can be seen in Figure 1a.

### 3.1. Nanoparticle Characterization

The morphology of the nanoparticles was studied using FESEM. As can be seen in Figure 1b–d, the images revealed that the nanoparticles showed not only a slight increase in size, but also higher aggregation after the autoclave treatment compared with the non-sterilized nanoparticles. It worth pointing out that nanoparticles irradiated at 5 kGy presented a higher homogeneity, with a rounded shape and a narrower size distribution. The morphology of the nanoparticles agrees with previously published research [32,33,34,35].

The hydrodynamic characterization of the nanoparticles dispersed in water, performed using DLS after the treatments, showed a progressive increase in the size of the nanoparticles from 140.8 ± 0.9 nm in the case of the non-sterilized nanoparticles, to 158.0 ± 1.6 nm in the case of the gamma irradiated nanoparticles at 10 kGy and reaching 164.3 ± 1.0 nm in the case of the autoclaved nanoparticles, as can be seen in Table 1.

The samples showed slight differences in their polydispersity values, as can be seen in Table 1. The gamma irradiated samples, irradiated at 5 or 10 kGy, presented the narrowest size distributions, followed by the non-sterilized, but without significant differences between them. Additionally, the autoclaved nanoparticles showed the highest significant values with respect to PdI, (*p* < 0.05). In the case of the zeta potential, only the autoclaving sterilizing treatment significantly affected the values of ζ, showing a significant decrease in the absolute value of ζ. There were no significant differences between the irradiated and the non-sterilized samples.

**Figure 2:**
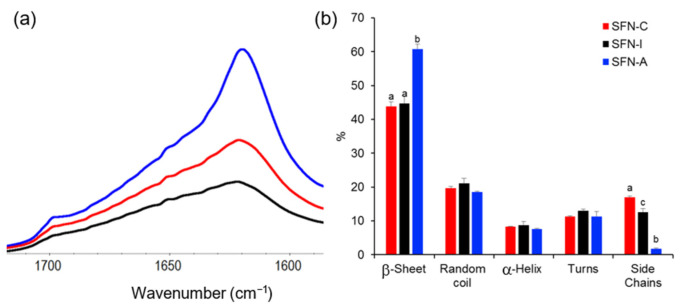
ATR-FTIR analysis of the secondary structure of the silk fibroin nanoparticles. (**a**) amide I region of the ATR-FTIR spectra of non-sterilized (SFN-C, red), gamma irradiated at 5 kGy (SFN-I, black), and autoclaved (SFN-A, blue) SFNs; (**b**) Secondary structure assignation obtained by Fourier self-deconvolution of the amide I peak. Different letters in the same group indicate significantly different subsets (*p* < 0.05).

Although the ATR-FTIR spectra of the sterilized samples showed the same profile of peaks than the non-sterilized sample measured as control in their full range (see Figure S1 of the Supplementary Materials), the amide I band of the autoclaved particles displayed a sharper peak at ~1620 cm^−1,^ as can be seen in Figure 2a. Figure 2b displays the proportion of different configurations of silk in the nanoparticles, which differ mainly in the β-sheet and side chain content.

The relative content of the secondary structures was determined by using the procedure described by Carissimi et al. [13], which is based on Fourier self-deconvolution and peak resolution. Evaluating the sample secondary structures, the β-sheet fraction of the autoclaved samples was significantly increased (60.8 ± 2.1%) compared with the non-sterilized (43.9 ± 1.2%) or the gamma irradiated nanoparticles (44.7 ± 1.4%).

Consequently, the autoclaved particles presented a significant reduction in their side chains fraction after the autoclave treatment (1.7 ± 1.0%) compared with those treated with 5 kGy (12.6 ± 0.2%) and the non-sterilized SFNs (17.0 ± 0.4%) (*p* < 0.05). The analysis also showed that the random coil, alpha helix, and turns fractions did not show significant variations post-sterilization. All the data can be consulted in the Table S1 of Supplementary Materials.

### 3.2. Stability Assays

**Figure 3:**
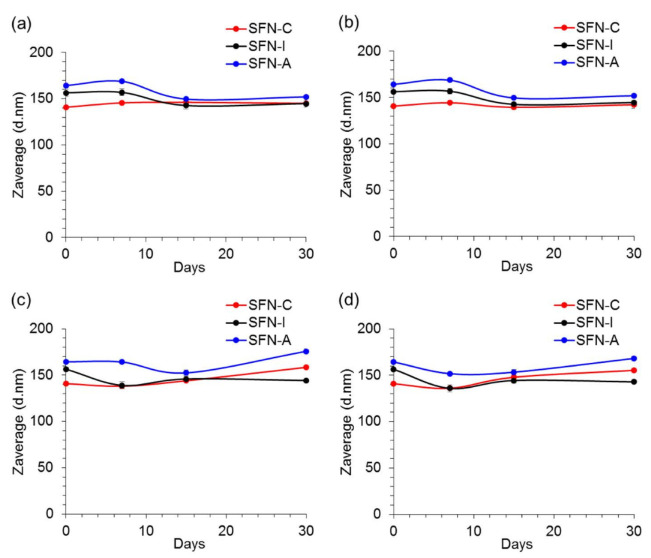
Effect of incubation temperature and aqueous media composition on the evolution of the hydrodynamic size (Z-average) of the non-sterilized (SFN-C, red), autoclaved (SFN-A, blue), and gamma irradiated at 5 kGy (SFN-I, black) nanoparticles for 30 days in: (**a**) Ultrapure water, 4 °C; (**b**) ultrapure water, 37 °C; (**c**) PBS 1× pH 7.4, 4 °C, and (**d**) PBS 1× pH 7.4, 37 °C. Values presented as Z-average (d.nm) ± SD (N = 9).

The stability of the nanoparticles during short-term storage was assessed via DLS measurements of the incubated samples (see Section 2.5 for the detailed experimental conditions). Neither the incubation temperature (at 4 °C or 37 °C) nor the composition of the solvent (ultrapure water or PBS 1× at pH 7.4) significantly affected the hydrodynamic size of the nanoparticles, as can be seen in Figure 3. Detailed values are presented in Table S2 of the Supplementary Materials.

When the nanoparticles were incubated in ultrapure water, the size of the particles was similar to their initial size (about 150 nm) after 30 days of incubation in both assayed temperatures. In the same way, the size of the nanoparticles incubated in PBS 1× pH 7.4 did not vary considerably along the assay in both temperatures.

In terms of the evolution of the PdI or ζ of the nanoparticles (see Figures S2 and S3 of the Supplementary Materials, respectively), neither the incubation temperature (4 °C or 37 °C) nor the composition of the solvent (ultrapure water or PBS 1× pH 7.4) affected the size of the nanoparticles.

**Figure 4:**
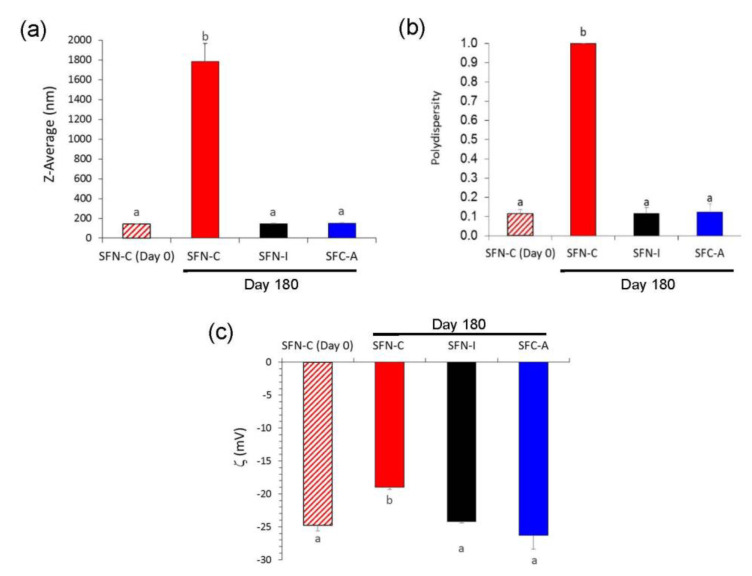
Effect of the time of storage on the hydrodynamic characteristics of the silk fibroin nanoparticles. (**a**) Z-average (nm); (**b**) polydispersity; (**c**) ζ (mV). Values presented as mean ± SD (N = 9). Different letters indicate significantly different values (*p* < 0.05).

In terms of long-term storage, i.e., for 180 days at 4 °C, changes in the hydrodynamic characteristics of the non-sterilized nanoparticles were more noticeable, as can be seen in Figure 4. SFN-C showed a shift in the values of ζ to lower absolute values, which produced a high aggregation due to the low electrostatic repulsion force, and thus increasing their Z-average to 1.786 ± 0.187 μm with a PdI of 1.000 ± 0.000. Although there were no visible changes in colour or aspect, the growth of microorganisms in the aqueous media produced an acidification of the solution to a pH of 5.5 and thus the partial protonation of the carboxylate groups of the fibroin and consequently a reduction in their absolute value of ζ. On the contrary, the sterilized nanoparticles, either via gamma irradiation (SFN-I) or autoclave treatment (SFN-A), maintained their hydrodynamic characteristics during storage for 180 days at 4 °C.

### 3.3. Microbiological Assays

The results showed that the samples treated via autoclaving or gamma irradiation with a dose equal or higher than 5 kGy, were efficiently sterilized. In the non-sterilized samples or the samples which were gamma irradiated at a low dose (<5 kGy), the growthof aerobic and facultative anaerobic microorganisms was detected, revealing that 5 kGy was the minimal dose necessary for the complete sterilization of the SFN aqueous suspensions. In the non-sterilized samples, *Sphingomonas paucimobilis* was detected, a persistent gram-negative nosocomial infectious organism. In the samples irradiated with 1 kGy, *Microbacterium oxydans*, a gram-positive bacterium from the genus of *Microbacterium* which occurs in human clinical specimens, and *Sphingomonas paucimobilis* were isolated. In the samples irradiated with 2.5 kGy, *Xophiala dermatitidis*, a dematiaceous fungus known to cause superficial, subcutaneous, cutaneous, and deep-seated infections, and, rarely, central line associated bloodstream infection, was isolated. All isolated and identified microorganisms included in the VITEK^®^ MS IVD database are common in hospital environments.

### 3.4. Cell Viability/Cytoxicity Assays

**Figure 5:**
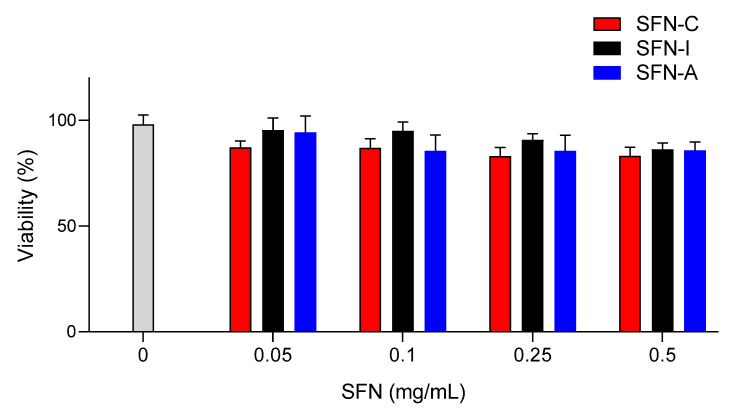
Cytotoxic effect of the non-sterilized (SFN-C, in red) or sterilized nanoparticles (SFN-I, in black and SFN-A, in blue) on murine fibroblast viability compared with the viability of L929 cells in absence of nanoparticles as control (100%). Data are expressed as percentage of cell viability ± SD vs. concentration. At least four samples per condition were measured.

The cytotoxic effect of the sterilized nanoparticles (SFN-I and SFN-A) on murine fibroblast viability was evaluated and compared with the non-sterilized nanoparticles (SFN-C). The selection of the L929 cell line for the cell viability/cytotoxicity assays was based on previously developed similar experiments [10,36], but the range of the concentrations assayed was increased to 0.5 mg/mL in order to cover future applications with a higher concentrations of nanoparticles. The mean value of the absorbance of the controls without nanoparticles was assumed to be 100% viability after 24 h of incubation (see Figure 5). No significant differences were found between the cell viability of the controls and that of the cells incubated with SFNs at concentrations in the range of 0.05–0.500 mg/mL.

## 4. Discussion

The sterilization of nanoparticles for biomedical applications is an essential stage in the preparation process and ensures a safe and stable product. Although sterilization methods are thoroughly described in the literature, validation is necessary since sterilization does not always guarantee sterility without altering the characteristics of the product. Thus, as a part of the development of SFNs as DDSs, we studied the effect of two types of sterilization processes on the characteristics of SFNs in aqueous solution.

The results showed that the samples which were either autoclaved or gamma irradiated with a dose equal or higher than 5 kGy were efficiently sterilized. The results from autoclaving were as expected, since it is a standard method for medicines in aqueous solutions [25] and it has been successfully applied in previous studies in relation to SF [22]. The results from gamma irradiation showed that sterility is reached at doses in the range of those described for polymeric nanoparticles by other authors [26]. These results are promising, since the possible effects of this irradiation on SFN characteristics are expected to be present at doses of 10 kGy or higher, as described for silver or chitosan nanoparticles by other authors [27,28]. In this way, it was shown that the nanoparticles sterilized using autoclaving presented a statistically significant increase in the content of β-sheet compared with the gamma irradiated or non-sterilized nanoparticles, something that had already been described by various authors [21,22]. This higher content of β-sheet could be responsible for the greater aggregation observed in the nanoparticles in both the FESEM images and in the DLS measurements, although it did not influence cell viability in the range of concentrations tested.

This slight increase in diameter or reduction in ζ observed for the autoclaved nanoparticles did not produce a significant change in their macroscopic characteristics, suspension stability, or cytotoxicity, but this could affect the ability of loading drugs or their internalization by the target cells in biomedical applications. However, the non-sterilized nanoparticles suspensions showed dramatic changes after 180 days of incubation. The growth of microorganisms acidified the suspensions to a pH of ~5.5, and consequently promoted the partial protonation of the carboxylate groups of the fibroin and a reduction in the absolute ζ values.

Although previous studies have shown greater cytotoxicity in irradiated silk biomaterials in melanoma tumour cells and how their immunomodulatory effects changed after treatment [18,19,37], 5 kGy irradiated SFNs did not show any difference in terms of viability with respect to the non-sterilized SFNs.

Radiation induced reactions are expected to occur through the radiolysis of water and the production of free radicals with high oxidative capacity, resulting in changes affecting the protein’s structure. These changes include degradation to smaller peptides or the aggregation of proteins [18]. According to previous studies, these reactions could lead to a positive effect on the physiological activities of silk irradiated at doses higher than 50 kGy [19,24,38].

The knowledge of the effects of sterilization on the physical and biological properties of the silk nanoparticles would allow the tailored production of SFNs as a function of their use. While autoclaving caused changes in the structural and physical properties of the nanoparticles (increasing size and β-Sheet content), gamma irradiation at 5 KGy is considered the most appropriate procedure if no changes in SFN characteristics are sought. If the enhancement of immunomodulation is the purpose, gamma irradiation with higher doses could be considered.

## 5. Conclusions

In conclusion, our intention with this work was to shed light on this crucial stage in the process of preparing SFNs. In this way, we have managed to reveal the changes that occur in the characteristics of the silk nanoparticles during sterilization. During this research, we have determined the minimum dose of gamma radiation necessary to effectively sterilize nanoparticles and its effects on the properties of nanoparticles. If particle sterility is relevant to a desired application, the fabrication of SFNs can be easily executed in a non-sterile environment, followed by a sterilization step, without compromising their properties. This is in comparison to other protein based biomaterials (e.g., collagen), which are sensitive to the autoclave sterilization process [38]. Finally, the structure and properties of the SFNs can be modulated by selecting the sterilization process according to the desired application. Further studies are necessaries in order to determine the effects of sterilization procedures of loaded nanoparticles with drugs or biomolecules for biomedical applications.

## Figures and Tables

**Table 1:** Hydrodynamic size or Z-average (d.nm), polydispersity index (PdI), and zeta potential (ζ, mV).

Sample	Z-Average (nm) ^1^	PdI ^1^	ζ (mV) ^1^
Non-sterilized	140.8 ± 0.9 ^a^	0.115 ± 0.019 ^a^	−24.8 ± 0.8 ^a^
1 kGy	153.6 ± 2.7 ^b^	0.117 ± 0.014 ^a^	−25.0 ± 0.7 ^a^
2.5 kGy	155.8 ± 1.9 ^b,c^	0.115 ± 0.024 ^a^	−24.7 ± 0.7 ^a^
5 kGy	156.4 ± 1.1 ^c^	0.103 ± 0.028 ^a^	−25.8 ± 1.0 ^a^
10 kGy	158.0 ± 1.6 ^c^	0.105 ± 0.012 ^a^	−25.9 ± 0.8 ^a^
Autoclaved	164.3 ± 1.0 ^d^	0.129 ± 0.012 ^b^	−22.0 ± 0.8 ^b^

^1^ Values presented as mean ± SD. N = 9. ^a–d^ Different uppercase letters in the same column indicate statistically significant differences between treatments (*p* < 0.05).

## Data Availability

The data presented in this study are available on request from the corresponding author.

## Supplementary Materials

The following supporting information can be downloaded at: https://www.mdpi.com/article/10.3390/polym14030498/s1, Figure S1: ATR-FTIR full spectra of the silk fibroin nanoparticles: non-sterilized (SFN-C, red), autoclaved (SFN-A, blue), and gamma irradiated at 5 kGy (SFN-I, black). Spectra re-scaled for a clearer visualization; Figure S2: Effect of incubation temperature and aqueous media composition on the evolution of the polydispersity index (PdI) of the non-sterilized (SFN-C, red), autoclaved (SFN-A, blue), and gamma irradiated at 5 kGy (SFN-I, black) nanoparticles for 30 days in: (a) Ultrapure water, 4 °C; (b) ultrapure water, 37 °C; (c) PBS 1× pH 7.4, 4 °C, and (d) PBS 1× pH 7.4, 37 °C. Values presented as PdI ± SD (N = 9); Figure S3: Effect of incubation temperature and aqueous media composition on the evolution of the ζ (mV) of the non-sterilized (SFN-C, red), autoclaved (SFN-A, blue), and gamma irradiated at 5 kGy (SFN-I, black) nanoparticles for 30 days in: (a) Ultrapure water, 4 °C; (b) ultrapure water, 37 °C; (c) PBS 1× pH 7.4, 4 °C, and (d) PBS 1× pH 7.4, 37 °C. Values presented as ζ (mV) ± SD (N = 9); Table S1: Silk fibroin secondary structure distribution (%) in the nanoparticles, determined by Fourier self-deconvolution and peak resolution; Table S2: Effect of incubation temperature (4 °C or 37 °C) and aqueous media composition (ultrapure water or PBS 1× pH 7.4) on the evolution of the hydrodynamic characteristics of the sterilized silk fibroin nanoparticles. (a) Ultrapure water, 4 °C; (b) ultrapure water, 37 °C; (c) PBS 1× pH 7.4, 4 °C; and (d) PBS 1× pH 7.4, 37 °C. Values presented as Mean ± SD (N = 9).
