# Supplementary material for: The Effect of Sterilization on the Characteristics of Silk Fibroin Nanoparticles
Source: Polymers (Basel). 2022 Jan 26;14(3):498. doi: 10.3390/polym14030498 (PMC8840090; doi:10.3390/polym14030498)
Supplement: Supplementary file 1 [file polymers-14-00498-s001.zip › polymers-1516575-supplementary.pdf]

polymers

MDPI

Supplementary Materials

The Effect of Sterilization on the Characteristics of Silk Fibroin Nanoparticles

María Alejandra Asensio Ruiz [1,2], Marta G. Fuster [3], Teresa Martínez Martínez [1,2], Mercedes G. Montalbán [3,*], José Luis Cenis [4], Gloria Víllora [3] and Antonio Abel Lozano-Pérez [2,4,*]

1 Unidad de Radiofarmacia, Hospital Clínico Universitario Virgen de la Arrixaca 30120, Murcia, Spain; mariaa.asensio@carm.es (M.A.A.R.); mteresa.martinez5@carm.es (T.M.M.)
2 Instituto Murciano de Investigación Biosanitaria (IMIB)-Arrixaca, 30120, Murcia, Spain
3 Chemical Engineering Department, Faculty of Chemistry, Regional Campus of International Excellence "Campus Mare Nostrum", University of Murcia, 30071 Murcia, Spain; marta.g.f@um.es (M.G.F.); gvillora@um.es (G.V.)
4 Departamento de Biotecnología, Genómica y Mejora Vegetal, Instituto Murciano de Investigación y Desarrollo Agrario y Medioambiental (IMIDA), 30150 Murcia, Spain; josel.cenis@carm.es (J.L.C.); abel@um.es (A.A.L.-P.)
* Correspondence: mercedes.garcia@um.es (M.G.M.); abel@um.es (A.A.L.-P.); Tel.: +34-86-888-7926 (M.G.M.); +34-96-836-8586 (A.A.L.-P.)

S.1. Nanoparticle characterization by ATR-FTIR.

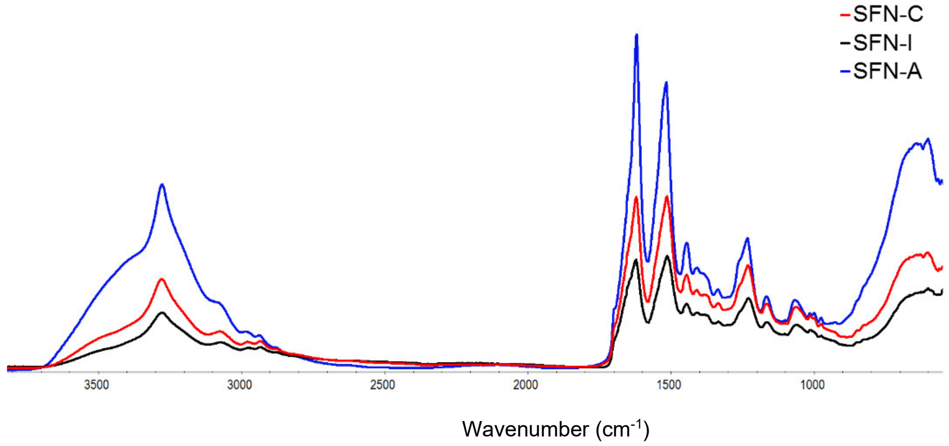

Figure S1. ATR-FTIR full spectra of the silk fibroin nanoparticles: non-sterilized (SFN-C, red), autoclaved (SFN-A, blue) and irradiated with 5 kGy (SFN-I, black). Spectra re-scaled for a clearer visualization.

Table S1. Silk fibroin secondary structure distribution (%) in the nanoparticles, determined by Fourier self-deconvolution and peak resolution.

Scheme 1.	Non-sterilized	Autoclaved	γ-Irradiated (5 kGy)
β-Sheet	43.9 ± 1.2[a]	60.8 ± 2.1[b]	44.7 ± 1.4[a]
Random coil	19.7 ± 0.4[a]	18.6 ± 1.6[a]	21.0 ± 0.2[a]
α-Helix	8.2 ± 0.2[a]	7.6 ± 1.0[a]	8.7 ± 0.1[a]
Turns	11.2 ± 0.4[a]	11.3 ± 0.6[a]	12.9 ± 1.4[a]
Side Chains	17.0 ± 0.4[a]	1.7 ± 1.0[b]	12.6 ± 0.2[c]

[1] Values presented as mean ± SD (N=3). [a-d] Different uppercase letters in the same row indicate statistically significant differences between treatments ($p < 0.05$).

S.2. Stability assays

Table 2. Effect of incubation temperature (4 °C or 37 °C) and aqueous media composition (ultrapure water or PBS 1x pH 7.4) on the evolution of the hydrodynamic characteristics of the sterilized silk fibroin nanoparticles. (**a**) Ultrapure water, 4 °C; (**b**) Ultrapure water, 37 °C; (**c**) PBS 1x pH 7.4, 4 °C and (**d**) PBS 1x pH 7.4, 37 °C. Values presented as Mean ± SD (N=9).

Days of incubation	Medium	Temperature	Sample	Z-Average (nm)	±SD	PdI	±SD	ζ (mV)	±SD
0	Water	-	Non-sterilized	140.8	0.9	0.115	0.019	−24.8	0.8
			1 kGy	153.6	2.6	0.117	0.014	−25.0	0.7
			2.5 kGy	155.8	1.9	0.115	0.024	−24.7	0.7
			5 kGy	156.4	1.1	0.103	0.028	−25.8	1.0
			10 kGy	158.0	1.6	0.105	0.012	−25.9	0.8
			Autoclave	164.3	0.9	0.129	0.012	−22.0	0.8
7	Water	4 °C	Non-sterilized	145.5	0.4	0.116	0.021	−24.7	0.2
			1 kGy	155.6	1.9	0.101	0.020	−25.1	0.8
			2.5 kGy	155.6	2.8	0.108	0.011	−26.3	0.8
			5 kGy	156.9	2.8	0.125	0.005	−26.0	0.6
			10 kGy	159.9	0.8	0.109	0.017	−26.5	0.9
			Autoclave	168.8	0.2	0.136	0.011	−22.2	0.4
		37 °C	Non-sterilized	144.4	0.8	0.114	0.016	−25.4	0.8
			1 kGy	156.2	0.8	0.111	0.012	−25.5	1.0
			2.5 kGy	159.2	1.5	0.103	0.015	−25.3	0.7
			5 kGy	157.8	1.5	0.122	0.005	−26.3	0.2
			10 kGy	158.9	0.5	0.120	0.018	−27.3	1.1
			Autoclave	171.0	1.1	0.130	0.011	−23.1	1.2
	PBS	4 °C	Non-sterilized	138.0	1.1	0.131	0.014	−24.2	1.5
			1 kGy	145.6	0.6	0.138	0.025	−23.3	1.2
			2.5 kGy	147.3	3.9	0.131	0.005	−22.9	1.4
			5 kGy	138.9	3.9	0.107	0.017	−26.2	1.1
			10 kGy	143.3	1.2	0.108	0.021	−25.4	0.4
			Autoclave	164.1	1.1	0.142	0.023	−21.5	1.5
		37 °C	Non-sterilized	135.9	3.4	0.115	0.031	−22.4	0.9
			1 kGy	136.6	1.7	0.108	0.014	−25.9	2.1
			2.5 kGy	135.9	0.9	0.136	0.018	−26.9	1.9
			5 kGy	135.8	0.9	0.123	0.012	−27.6	2.2
			10 kGy	137.8	2.0	0.119	0.017	−25.0	0.9
			Autoclave	151.7	2.6	0.133	0.041	−23.7	1.9
15	Water	4 °C	Non-sterilized	146.1	0.5	0.105	0.025	−23.7	0.3
			1 kGy	144.3	1.6	0.114	0.020	−22.8	0.5
			2.5 kGy	142.4	1.6	0.121	0.01	−24.0	0.4
			5 kGy	142.7	1.6	0.118	0.016	−24.4	1.3
			10 kGy	137.8	0.7	0.133	0.017	−26.0	1.8
			Autoclave	149.6	0.6	0.144	0.019	−21.8	0.9
		37 °C	Non-sterilized	139.5	2.2	0.127	0.013	−21.7	0.8
			1 kGy	137	3.0	0.138	0.015	−21.3	1.5
			2.5 kGy	143.7	1.4	0.114	0.013	−22.5	0.9
			5 kGy	134.8	1.4	0.112	0.011	−22.2	0.4
			10 kGy	140.8	0.8	0.13	0.03	−23.8	1.0
			Autoclave	152.1	1.7	0.137	0.017	−19.8	0.8
	PBS	4 °C	Non-sterilized	143.9	1.1	0.117	0.011	−26.0	0.2
			1 kGy	143.2	1.0	0.132	0.011	−25.6	0.7
			2.5 kGy	144.4	2.6	0.131	0.011	−25.9	0.4
			5 kGy	145.8	2.6	0.131	0.014	−25.9	0.9
			10 kGy	139.2	1.1	0.131	0.005	−26.9	0.3
			Autoclave	152.4	2.9	0.156	0.007	−23.4	0.5
		37 °C	Non-sterilized	147.8	0.7	0.133	0.029	−21.0	1.3

			1 kGy	145.1	1.9	0.138	0.024	−25.2	1.8
			2.5 kGy	150.1	2.8	0.167	0.03	−24.3	1.3
			5 kGy	144.3	2.8	0.135	0.001	−23.8	1.5
			10 kGy	142.1	2.3	0.155	0.003	−23.4	0.8
			Autoclave	153.3	4.2	0.156	0.013	−21.0	1.1
30	Water	4 °C	Non-sterilized	145	1.5	0.124	0.016	−24.6	1.4
			1 kGy	142.1	1.0	0.104	0.007	−24.1	1.1
			2.5 kGy	143.3	1.6	0.107	0.015	−25.5	0.9
			5 kGy	144.7	1.6	0.118	0.013	−26.0	0.4
			10 kGy	146.6	0.7	0.12	0.023	−26.0	0.6
			Autoclave	152	1.9	0.129	0.004	−22.7	1.0
		37 °C	Non-sterilized	142.3	3.7	0.136	0.006	−23.8	0.3
			1 kGy	142.9	0.8	0.132	0.011	−26.4	0.8
			2.5 kGy	144.1	1.2	0.128	0.01	−25.5	0.4
			5 kGy	145.4	1.2	0.118	0.013	−26.2	0.8
			10 kGy	146.4	0.5	0.107	0.013	−27.2	0.4
			Autoclave	151.5	1.5	0.15	0.016	−22.0	0.8
	PBS	4 °C	Non-sterilized	158.4	1.7	0.149	0.018	−21.4	1.3
			1 kGy	142.9	1.3	0.124	0.024	−24.6	1.1
			2.5 kGy	142.2	1.6	0.115	0.029	−23.9	1.1
			5 kGy	144.2	1.6	0.134	0.005	−23.9	1.5
			10 kGy	141.7	1.4	0.104	0.003	−24.9	1.2
			Autoclave	175.7	2.5	0.164	0.016	−20.6	1.3
		37 °C	Non-sterilized	155.3	1.8	0.132	0.008	−21.9	0.6
			1 kGy	146	0.9	0.11	0.029	−24.4	0.9
			2.5 kGy	138.9	1.9	0.114	0.02	−25.6	1.4
			5 kGy	142.9	1.9	0.119	0.015	−25.1	2.1
			10 kGy	143.2	1.0	0.111	0.026	−25.4	2.0
			Autoclave	168.1	4.0	0.138	0.016	−21.9	1.1

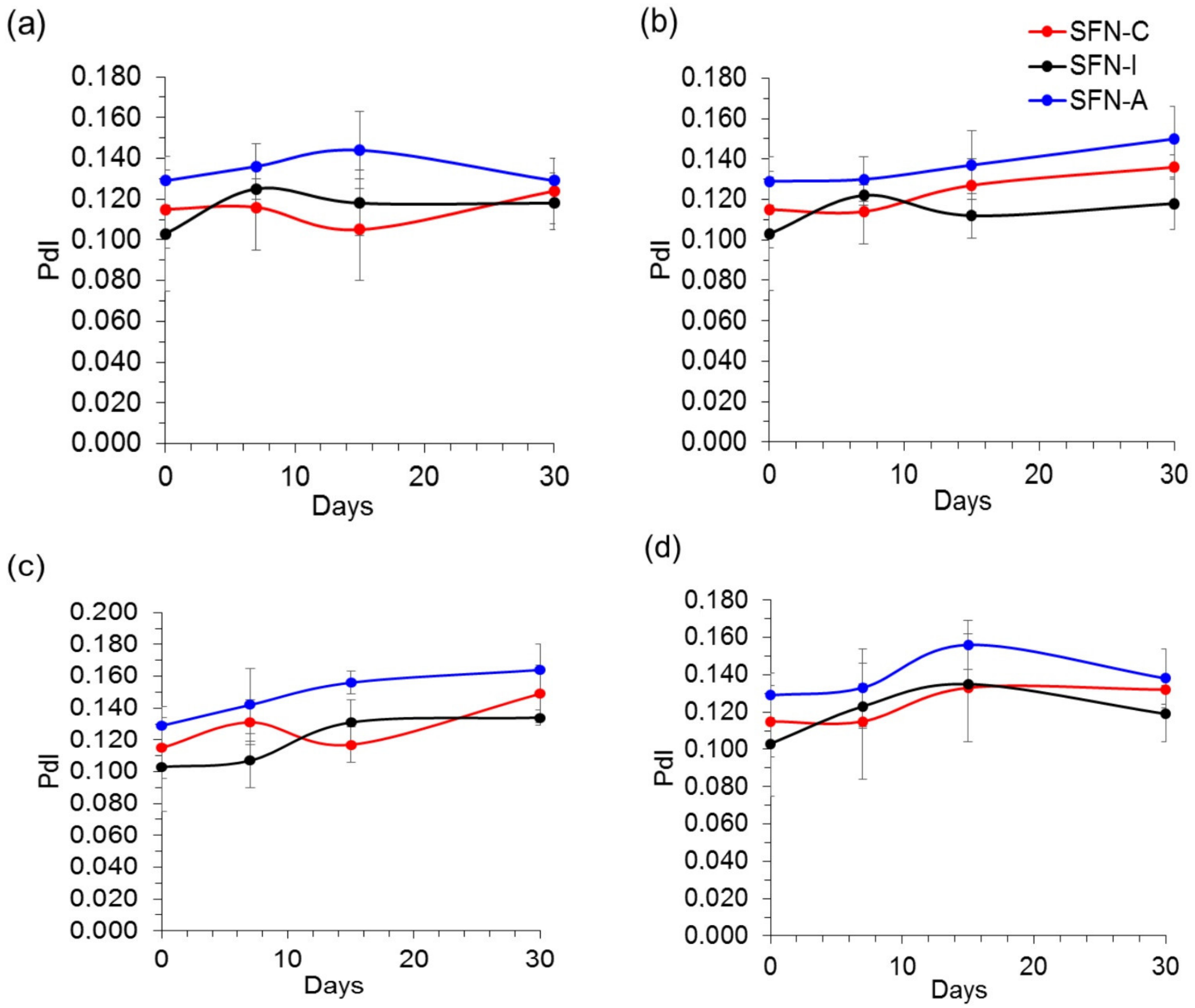

Figure S2. Effect of incubation temperature and aqueous media composition on the evolution of the Polydispersity Index (PdI) of the non-sterilized nanoparticles (SFN-C, red), autoclaved (SFN-A, blue) and γ-irradiated with 5 kGy (SFN-I, black) for 30 days in: (**a**) Ultrapure water, 4 °C; (**b**) Ultrapure water, 37 °C; (**c**) PBS 1x pH 7.4, 4 °C and (**d**) PBS 1x pH 7.4, 37 °C. Values presented as PdI ± SD (N=9).

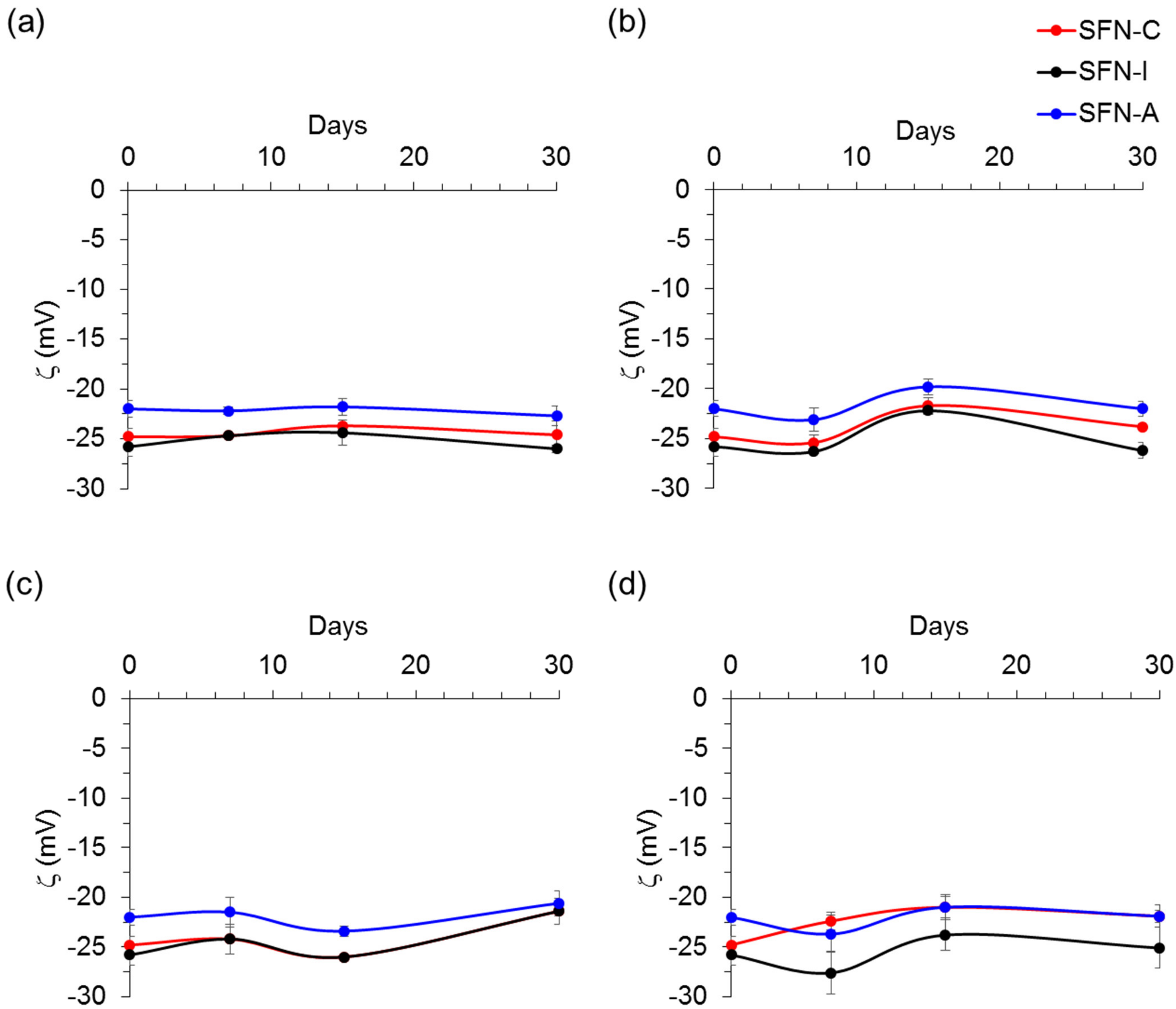

Figure S3. Effect of incubation temperature and aqueous media composition on the evolution of the ζ (mV) of the non-sterilized nanoparticles (SFN-C, red), autoclaved (SFN-A, blue) and γ-irradiated with 5 kGy (SFN-I, black) for 30 days in: (**a**) Ultrapure water, 4 °C; (**b**) Ultrapure water, 37 °C; (**c**) PBS 1x pH 7.4, 4 °C and (**d**) PBS 1x pH 7.4, 37 °C. Values presented as ζ (mV) ± SD (N=9).
